# Information theoretic approaches for inference of biological networks from continuous-valued data

**DOI:** 10.1186/s12918-016-0331-y

**Published:** 2016-09-06

**Authors:** David M. Budden, Edmund J. Crampin

**Affiliations:** 1 Massachusetts Institute of Technology, Computer Science and Artificial Intelligence Laboratory, Cambridge, 02139 USA; 2 Systems Biology Laboratory, Melbourne School of Engineering, the University of Melbourne, Parkville, 3010 Australia; 3 ARC Centre of Excellence in Convergent Bio-Nano Science and Technology, Parkville, 3010 Australia; 4 Department of Mathematics and Statistics, the University of Melbourne, Parkville, 3010 Australia; 5 School of Medicine, the University of Melbourne, Parkville, 3010 Australia

**Keywords:** Gene regulatory network, Transcriptional regulation, Gene expression

## Abstract

**Background:**

Characterising programs of gene regulation by studying individual protein-DNA and protein-protein interactions would require a large volume of high-resolution proteomics data, and such data are not yet available. Instead, many gene regulatory network (GRN) techniques have been developed, which leverage the wealth of transcriptomic data generated by recent consortia to study indirect, gene-level relationships between transcriptional regulators. Despite the popularity of such methods, previous methods of GRN inference exhibit limitations that we highlight and address through the lens of information theory.

**Results:**

We introduce new model-free and non-linear information theoretic measures for the inference of GRNs and other biological networks from continuous-valued data. Although previous tools have implemented mutual information as a means of inferring pairwise associations, they either introduce statistical bias through discretisation or are limited to modelling undirected relationships. Our approach overcomes both of these limitations, as demonstrated by a substantial improvement in empirical performance for a set of 160 GRNs of varying size and topology.

**Conclusions:**

The information theoretic measures described in this study yield substantial improvements over previous approaches (e.g. ARACNE) and have been implemented in the latest release of NAIL (Network Analysis and Inference Library). However, despite the theoretical and empirical advantages of these new measures, they do not circumvent the fundamental limitation of indeterminacy exhibited across this class of biological networks. These methods have presently found value in computational neurobiology, and will likely gain traction for GRN analysis as the volume and quality of temporal transcriptomics data continues to improve.

## Background

Although it is well-established that networks of molecular interactions underlie critical cellular functions including development, differentiation and homeostasis, accurate reconstruction of network topologies using only gene expression data is a difficult problem that has received much attention in recent years [1–3]. Gene regulatory networks (GRNs) assume that active regulatory interactions can be captured as weighted, pairwise associations between genes and, accordingly, that complex interactions (e.g. between RNAs and proteins) may be mapped onto this level.

In 2007, the Dialogue for Reverse Engineering Assessments and Methods (DREAM) challenge was launched to promote and advance research in network-based analyses of biological data [4, 5]. Early DREAM challenges focused primarily on simulated data-sets, whereby a ‘true’ network topology was used to generate artificial gene expression data [6, 7]. Entrants developed algorithms to reconstruct this network from the expression data alone, with performance evaluated empirically against the supposed true network. Subsequent DREAM challenges have introduced experimental data, with mRNA transcript abundance quantified using qPCR (quantitative polymerase chain reaction), microarray and now high-throughput RNA-seq technologies. Although more biologically relevant than simulated data, ‘true’ network topologies for these systems remain fragmentary approximations of gene regulatory interactions and are thus inappropriate for benchmarking [5].

The University of Melbourne’s Systems Biology Laboratory has previously developed software (NAIL, Network Analysis and Inference Library [8, 9]) for inferring, analysing and visualising GRNs to assist with our efforts in transcriptional regulatory modelling [9–13]. As noted in a recent review by Novère [14], NAIL integrates several previously-published methods of GRN analysis. Most of these approaches apply a variant of Pearson’s correlation coefficient to assign edge-weights to each pair of genes, *X* and *Y*:

$$r_{X,Y} = \frac{E[(X-\hat{\mu}_{X})(Y-\hat{\mu}_{Y})]}{\hat{\sigma}_{X}\hat{\sigma}_{Y}}, $$

where $\hat {\mu }_{X}$ and $\hat {\sigma }_{X}$ are the sample mean and standard deviation of *X*, respectively. Due to the nature of the GRN evaluation metrics (described later), arbitrary thresholds do not need to be assigned to identify edge weights accepted as representing physical biological interactions.

Although correlation is a straightforward method for assigning network edge weights, it has several fundamental limitations. Firstly, Pearson’s *r* assumes that *X* and *Y* are normally-distributed and thus it can only identify linear relationships, which can be unsuitable in the context of qPCR, microarray or RNA-seq-quantified transcript abundance. Rank-based correlation metrics such as Spearman’s *ρ* and Kendall’s *τ* coefficients are often applied to partially correct for this issue. Secondly, correlation is a symmetric measure (*r*_*X,Y*_=*r*_*Y,X*_) and thus it can not infer the directionality or causality of biological interactions, even when applied to the analysis of appropriate time-series or gene knock-down data.

Despite widespread practical utility of GRN analysis, recent studies have made broad-stroked dismissals of the field on theoretical bases [15]. Instead, we take a two-fold approach of (a) building upon previous inference methods by leveraging the latest information theoretic advancements, and (b) discussing alternative modelling approaches that better suit some scenarios. Our measures have been implemented in post-publication versions of NAIL [8, 9], and we refer the reader to [16] for our general commentary on the conundrum of reconciling theoretical versus empirical performance bounds.

## Methods

This section describes how recent innovations in information theory may be effectively applied to infer network connectivity from continuous-valued biological data. Although described in the context of GRN inference, the following techniques can be applied to many other domains (e.g. inference of neural, proteomic or metabolomic networks).

### Information theoretic measures for biological network inference

The fundamental measure of information theory is Shannon entropy [17] (measured in bits), which captures the expected uncertainty associated with any measurement, *x*, of a random variable, *X*:

$$H(X) = -\sum\limits_{x\in X} p(x)\log_{2}(p(x)), $$

where *p*(*x*) is the (marginal) probability distribution of *X*. In order to model continuous-valued mRNA transcript levels, it is necessary to instead consider the closely-related differential entropy of *X* (measured in nats) [18]:

$$H_{D}(X) = -\int_{X} f(x)\log(f(x))~dx, $$

where *f*(*x*) is the probability density function (PDF) of *X*, *f*(*x*)>0. This definition can be extended to quantify the joint entropy of *X* and *Y*:

$$H_{D}(X,Y) = -\iint_{D} f(x,y)\log(f(x,y))\,dx\,dy, $$

where *f*(*x,y*) is the joint PDF of *X* and *Y*, and *D*⊆*X*×*Y* such that the marginal and joint PDFs of *X* and *Y* are strictly positive. The mutual information of *X* and *Y* can then be defined in terms of these two measures:

$$\begin{array}{*{20}l} I_{D}(X; Y) &= H_{D}(X) + H_{D}(Y) - H_{D}(X,Y)\\ &= -\iint_{D} f(x,y)\log \left(\frac{f(x,y)}{f(x)f(y)} \right)\,dx\,dy, \end{array} $$

which is interpreted as the symmetric quantity of information ‘shared’ by *X* and *Y*, *I*_*D*_(*X;Y*)=*I*_*D*_(*Y;X*). Mutual information makes no assumptions regarding the distribution or linearity of relationships between transcript abundance values.

Mutual information (MI) has been applied to assigning edge weights in previous GRN studies, with MI-based network inference tools including minet [19], relevance networks [20], MRNET [21] and earlier iterations of NAIL [8]. However, these tools have generally applied variations of binning algorithms to discretise X and Y and allow for the calculation of discrete mutual information:

$$I(X; Y) = \sum\limits_{x\in X}\sum\limits_{y\in Y}\hat{p}(x,y)\log_{2}\left(\frac{\hat{p}(x,y)}{\hat{p}(x)\hat{p}(y)} \right), $$

where $\hat {p}(x)$ and $\hat {p}({x,y})$ are predictors of the marginal and joint distributions of *X* and *Y*, typically implemented as simply the empirical (sampled) distribution of gene expression values. Despite the introduction of several bias-correction techniques [22–24] and the observation that $I_{D}({X;Y}) = {\lim }_{\Delta \to 0}I(X^{\Delta }; Y^{\Delta })$ for discretisations *X* ^*Δ*^ and *Y* ^*Δ*^ with bin width *Δ* [25], it is well-established that discretisation is a suboptimal method for handling empirical distributions of continuous-valued data [26–28]. Although earlier studies have proposed continuous estimation schemes for gene expression data, the focus has been on temporal interpolation (i.e. correcting for non-uniform *sampling* or missing observations [29]) rather than the *quantization* error introduced by previous information theoretical approaches. In the following sections we propose and describe several methods of continuous MI estimation that specifically address the latter class of errors.

### Mutual information estimators for continuous-valued data

The simplest method of continuous-valued MI estimation is the Gaussian distribution model, under which multivariate joint entropy can be expressed as [25]:

$$H(\mathbf{X}) = \frac{1}{2}\log\left((2\pi e)^{N} |\Sigma_{\mathbf{X}}| \right), $$

where $\mathbf {X} \in \mathcal {R}^{M\times N}$ is the matrix of *M* expression values for *N* genes, and *Σ*_**X**_ is the covariance matrix of **X**. The pairwise MI between genes *X* and *Y* can then be calculated as *I*_*D*_(*X; Y*)=*H*(*X*)+*H*(*Y*)−*H*(*X,Y*). Although this approach is computationally efficient and extends well to large GRNs, it reintroduces the assumption of normally-distributed and linearly-associated variables and is thus inappropriate for modelling qPCR, microarray or RNA-seq gene expression data.

An alternative to the Gaussian distribution model is to estimate the marginal and joint PDFs of **X** using a kernel function, *K*(·):

1 $$  \hat{f}(\mathbf{X}) = \frac{1}{Mh}\sum\limits_{i=1}^{M} K\left(\frac{\mathbf{X}-X_{i}}{h}\right),  $$

where *h* is the kernel bandwidth and *X*_*i*_ is the *i*-th row of **X**; e.g. 〈*X*_*i*_;*Y*_*i*_〉 for the calculation of pairwise MI between genes *X* and *Y*. Two methods of kernel estimation have been introduced into the latest version of NAIL [9], including the uniform kernel:

2 $$  K(\mathbf{X}) = \frac{1}{2}\mathbf{1}_{\{|\mathbf{X}| \leq 1\}},  $$

where **1**_{·}_ is the indicator function; and the Gaussian kernel, as implemented in the popular ARACNE package [30]:

$$ K(\mathbf{X}) = (2\pi)^{N/2}\exp\left(-\frac{1}{2}\mathbf{X}^{\top} \mathbf{X}\right). $$

Unlike the Gaussian distribution model, kernel density estimation allows for the model-free identification of non-linear relationships between gene expression levels. However, it is both statistically biased and sensitive to the selection of kernel bandwidth [31, 32]. To provide a bias-corrected and robust method for continuous-valued MI estimation, we instead implement and evaluate two variants of the Kraskov, Stögbauer and Grassberger (KSG) algorithm [33].

For each matched observation 〈*x*_*m*_;*y*_*m*_〉 of genes *X* and *Y*, the first KSG algorithm calculates the difference in expression, 〈*Δ*_*x*_;*Δ*_*y*_〉, between that observation and its *K*-th nearest neighbour in *X*×*Y*. The number of neighbours within max{*Δ*_*x*_,*Δ*_*y*_} of *x*_*m*_ and *y*_*m*_ are then calculated in their respective marginal spaces, with *n*_*x*_ and *n*_*y*_ defined as the mean of these counts across all matched observations. The MI of genes *X* and *Y* can then be estimated using the first KSG algorithm:

3 $$  I_{D}^{(1)}(X; Y) = \psi(K) + \psi(M) - (\psi(n_{x} + 1) + \psi(n_{y} + 1)),  $$

where *ψ*(·) is the digamma function:

$$\psi(x) = \frac{d}{dx}\log\Gamma(x) = \frac{\Gamma^{\prime}(x)}{\Gamma(x)}. $$

The second KSG algorithm calculates *n*_*x*_ and *n*_*y*_ by considering *Δ*_*x*_ and *Δ*_*y*_ separately (rather than their maximum), yielding the following alternative MI estimator:

4 $$  I_{D}^{(2)}(X; Y) = \psi(K) + \psi(M) - \frac{1}{K} - (\psi(n_{x}) + \psi(n_{y})),  $$

which is more accurate for large values of *M* and thus more appropriate for large (genome-wide) GRN inference. Both of these algorithms correct for bias and have been empirically demonstrated as robust to the selection of *K* [33].

### Extensions to information theoretic network inference

Despite MI providing a non-linear and model-free approach for quantifying pairwise associations between genes, it suffers from another fundamental limitation common of correlation-based analysis: spurious inference of fully-connected subgraphs (*K*-vertex cliques) caused by indirect regulation of the form *g*_1_⋯⇔*g*_2_⇔⋯⇔*g*_*K*_. Under the simplified assumption that genes *g*_1_ and *g*_*K*_ only interact via a single path through *g*_2_, these cliques can be reduced to a single pathway by considering the data processing inequality (DPI) [34]:

$$I_{D}(g_{1}; g_{K}) \leq \min\{I_{D}(g_{1}; g_{2}), I_{D}(g_{2}; g_{K}) \}, $$

which can be expressed concisely as ‘post-processing cannot increase information’ (colloquially, ‘garbage in, garbage out’). The DPI was first leveraged in the context of GRN inference by ARACNE [30], where it reduces connectivity of the final boolean connectivity matrix (obtained by applying a minimum MI threshold to edge weights) by removing the lowest-MI edge for all fully-connected gene triplets.

Although the DPI is proven to reconstruct correct network topology given the aforementioned assumptions [30], the existence of a single isolated pathway of regulation between any pair of genes is known to be an over-simplification of most biological regulatory processes (exemplified by signaling pathway cross-talk [35, 36]). We instead propose an alternative method of resolving GRN cliques that involves calculating MI between the transcript abundances of two genes, *X* and *Y*, conditioned on abundance of a third gene, *Z*:

$${}I_{D}(X;Y|Z) = \iiint_{T} f(x,y,z)\log\left(\frac{f(x,y,z)f(z)}{f(x,z)f(y,z)}\right)\,dx\,dy\,dz, $$

where *T*⊆*X*×*Y*×*Z* such that all marginal and joint PDFs are strictly positive. Importantly, the conditional MI between *X* and *Y* given *Z* can be either smaller or larger than *I*_*D*_(*X; Y*); conditioning removes redundant information between *Y* and *Z* regarding *X*, but identifies synergistic information that requires both *Y* and *Z* to be known. In the earlier example of indirect regulation of the form *g*_1_⋯⇔*g*_2_⇔⋯⇔*g*_*K*_, a strong MI between *g*_1_ and *g*_*K*_ conditioned on *g*_2_ would indicate additional regulatory pathways between *g*_1_ and *g*_*K*_ that do not involve *g*_2_. In such scenarios, application of the DPI would be inappropriate.

Conditional MI can be estimated for continuous-valued data using a similar technique. For kernel estimation, this simply involves applying Eq. (1) to approximate the marginal PDF, *f*(*z*), and joint PDFs, *f*(*x,y,z*), *f*(*x,z*) and *f*(*y,z*). The KSG algorithms can also be applied in the following form [37, 38]:

$${\begin{aligned} I^{(1)}_{D}(X;Y|Z) &= \psi(K) + \psi(n_{z} + 1) - \psi(n_{xz} + 1) - \psi(n_{yz} + 1)\\ I^{(2)}_{D}(X;Y|Z) &= \psi(K) - \frac{2}{K} + \psi(n_{z}) - \psi(n_{xz}) + \frac{1}{n_{xz}} -\psi(n_{yz}) + \frac{1}{n_{yz}}, \end{aligned}} $$

where *n*_*xz*_ and *n*_*yz*_ refer to counts in *X*×*Z* and *Y*×*Z* respectively.

If one has access to uniformly-sampled time series of transcript abundance data *X*={…,*X*_*n*−1_,*X*_*n*_,*X*_*n*+1_,… } (and likewise for *Y*), the directional transfer of information from *X* to *Y* can be determined by conditioning their MI on past observations of *Y*. For a Markovian process of length *k*, the transfer entropy from *X* to *Y* is this defined as [32, 39]:

$$T_{X\to Y} = I_{D}(X_{n+1}; Y_{n+1} | Y_{n-k:n}), $$

where *Y*_*n*−*k:n*_ is the length-*k* history of *Y* preceding time *n*. This definition generalises to non-Markovian processes for ${\lim }_{k\to \infty }$ and can be extended to calculate pairwise information transfer conditioned on a third gene (or set of genes), *Z*:

$$T_{X\to Y|Z} = I_{D}(X_{n+1}; Y_{n+1} | Y_{n-k:n}, Z). $$

Transfer entropy (TE) allows directional gene regulatory associations to be inferred, which can be interpreted as capturing evidence of causal relationships. Under the erroneous assumption that gene expression values are normally distributed, TE reduces to Granger Causality [40], which has previously been applied to sparse vector autoregressive (SVAR) inference of GRNs from microarray data [41].

### Data and evaluation

**Fig. 1:**
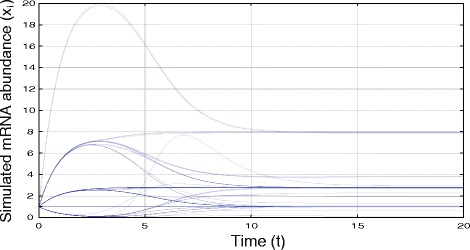
Transcriptional activity of each gene in a Century-series (100 node) scale-free Mendes network [42], simulated using multiplicative Hill kinetics as defined in 5. Each time-series was simulated until convergence (*d* *x*/*d* *t*=0) using Gepasi [45], from which gene-level correlation, MI or TE can be calculated for GRN approximation

Network inference from pairwise MI and TE was evaluated on the benchmark collection of synthetic GRNs proposed by Mendes et al. [42]. Although synthetic data is not ideal for validation, there are very few (if any) biological systems studied in sufficient detail for confident assertions regarding their true underlying topology [5]. Instead, the ‘gold standard’ Mendes models apply a simplified, bottom-up model (multiplicative Hill kinetics [43, 44], simulated in Gepasi [45]) to approximate transcriptional activity, as illustrated in Fig. 1:

5 $$  \frac{dx_{i}}{dt} = \alpha_{i}\prod_{j=1}^{N_{I}}\frac{IK_{j}^{n_{j}}}{IK_{j}^{n_{j}} + I_{j}^{n_{j}}} \prod_{\ell=1}^{N_{A}}\left(1 + \frac{A_{\ell}^{m_{\ell}}}{AK_{\ell}^{m_{\ell}} + A_{\ell}^{m_{\ell}}} \right)- \beta_{i} x_{i},  $$

where *x*_*i*_ is the ‘concentration’ of gene product *i*, *N*_*A*_ and *N*_*I*_ are the number of activators and inhibitors (with concentrations *A* and *I*) and *K* represents the concentration at which activating/inhibiting effects are half their saturated value. The efficiency of mRNA transcription and degradation for the *i*-th gene are parameterised by *α*_*i*_ and *β*_*i*_ respectively, and *n* controls the sigmoidicity of the function.

**Fig. 2:**
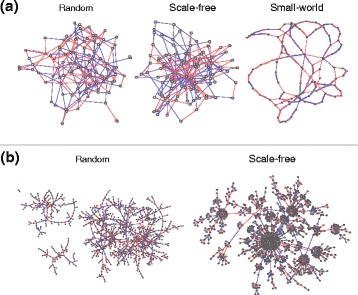
Examples of the Mendes synthetic GRNs used to benchmark the performance of the information theoretic measures proposed in this article [42], with *blue* and *red* edges representing activating and inhibiting interactions respectively. Erdős-Rényi [46] (random), Watts-Strogatz [47] (small-world) and Albert-Barabási [48] (scale-free) topologies were considered from both the (**a**) ‘Century’ (100-node) and (**b**) ‘Jumbo’ (1000-node) series. Of these topologies, there is growing evidence that scale-free networks most accurately represent the organisation of metabolic and transcriptomic regulatory systems [49–51]

The Mendes models allow benchmarking across several classes of network topologies, including including Erdős-Rényi [46] (random), Watts-Strogatz [47] (small-world) and Albert-Barabási [48] (scale-free) networks (examples provided in Fig. 2). As there is growing evidence that scale-free networks are appropriate for describing metabolic and transcriptomic interactions [49–51], 50 ‘Century’ (100-node) and 5 ‘Jumbo’ (1000-node) scale-free Mendes networks are considered [42], along with a variety of random and small-world topologies.

The performance of each inferred GRN is evaluated using the Area Under the receiver operator characteristic (ROC) Curve (AUC) metric [52]:

$$AUC = \frac{1}{2}\sum\limits_{k-1}^{n} (X_{k} - X_{k-1})(Y_{k} + Y_{k-1}), $$

where *X*_*k*_ is the false positive rate and *Y*_*k*_ is the true positive weight for the *k*-th output in the ranked list of predicted edge weights; i.e. the AUC is the trapezoidal Riemann sum of the ROC curve.

## Results and discussion

In this Section we compare the performance of the directed and undirected information theoretic measures described, using the gold-standard Mendes suite of benchmark GRNs [42]. For kernel-based MI estimation we use the popular ARACNE implementation, the results of which are consistent with our implementation in NAIL [9]. Average performances (AUC) and standard deviations are presented in Table 1.

**Table 1:** Performance of MI and TE-based methods of GRN inference, presented as the mean AUC (and standard deviation) across a variety of random [46], small-world [47] and scale-free [48] networks from the Mendes ‘Century’ and ‘Jumbo’ collections [42]

Collection	Networks	Nodes	Edges	Topology	AUC (Mutual Information)		AUC (Transfer Entropy)	
					Kernel (ARACNE [30])	KSG	Kernel	KSG
CenturyRND	50	100	200	Random	0.514	0.478	0.589	0.603
					(0.030)	(0.028)	(0.024)	(0.027)
CenturySF	50	100	200	Scale-free	0.475	0.505	0.526	0.561
					(0.036)	(0.033)	(0.030)	(0.030)
CenturySW	50	100	200	Small-world	0.477	0.471	0.602	0.598
					(0.035)	(0.035)	(0.028)	(0.030)
JumboRND	5	1000	1000	Random	0.473	0.439	0.540	0.564
					(0.014)	(0.013)	(0.006)	(0.009)
JumboSF	5	1000	1000	Scale-free	0.526	0.577	0.606	0.649
					(0.007)	(0.010)	(0.007)	(0.012)

Kernel-based methods apply the uniform kernel (see (2)) with bandwidth *h*=0.1. For KSG-based methods, KSG algorithm 1 (better suited to small networks, see (3)) was applied to ‘Century’ data and algorithm 2 (see (4)) to ‘Jumbo’ data, both with *K*=4 [33] and assuming length-1 Markovian processes. Gene expression time-series were simulated until convergence (*d* *x*/*d* *t*=0) using Gepasi with default parameters [45]

It is evident that the performance of MI-based inference of undirected GRNs is comparable to random guessing (Taxble 1, theoretic *A* *U* *C*=0.5). Application of the DPI yielded no significant improvement for any MI estimator. These results are consistent with recent studies which found that the most sophisticated GRN algorithms perform no better than simple correlation-based inference, due to the fundamental limitation of considering only pairwise expression relationships. A detailed analysis by Maetschke et al. demonstrated that the utility of these techniques is limited to small networks with star-like topologies and that exclusively contain activating or inhibiting interactions [11]. Several common regulatory network motifs have since been identified that are particularly difficult to infer [30]. Moreover, Krishnan et al. have provided a theoretical explanation as to why many non-trivial GRNs are unable to be reverse-engineered from expression data alone; i.e. multiple dissimilar networks produce indistinguishable abundance profiles due to latent protein-mediated effects [15].

The inclusion of directed information transfer to extend GRN inference yielded improved performance across all networks, with all TE-based methods performing significantly better than random (presumably because these measures are better able to capture activation and inhibition relationships, which are inherently directional). These methods outperformed other Mendes-benchmarked algorithms applying variants of correlation or MI estimation [11, 20, 30], and thus both kernel and KSG-estimated TE have been implemented for causal network inference in the latest version of NAIL [9]. To our knowledge, this is the most comprehensive set of information theoretic tools available for biological network inference. NAIL is available to download from https://sourceforge.net/projects/nailsystemsbiology/.

## Conclusions

Previous GRN inference frameworks have implemented mutual information as a means of inferring pairwise gene-level associations (e.g. minet [19], relevance networks [20], MRNET [21] and ARACNE [30]). However, these tools either introduce statistical bias through discretisation of expression data or are limited to modelling undirected relationships. In this article, we have proposed and evaluated new model-free and non-linear information theoretic measures that circumvent these limitations, leading to substantial improvement in empirical performance across a benchmark set of 160 synthetic GRNs.

Although NAIL is the first GRN toolkit to incorporate the measures described in this article, it does not overcome another fundamental limitation of previous models; i.e. unambiguous network reconstruction requires that the number of time samples must be greater than the number of genes, and even the highest time resolution data-sets fall short by several orders-of-magnitude. To explore transcriptional regulation in the context of current data availability, we refer the reader to the emerging body of literature surround predictive gene expression modelling [53–55]. This class of top-down modelling leverages transcriptomic and epigenetic data as independent observations of an underlying regulatory function, thus circumventing the issue of indeterminacy inherent to GRN analysis.

Despite conflicting reports of the utility of GRNs between theoretical and empirical studies [16], we believe that this class of network inference will continue to be of widespread value for exploring fundamental regulatory processes. Moreover, the methods described in this paper can be readily applied to computational neuroscience [56, 57] and other fields of complex systems theory [58, 59]. We encourage researchers to investigate how such network abstractions can be applied to their class of biological problems.
